# Inhibition of miR-185-3p Confers Erlotinib Resistance Through Upregulation of PFKL/MET in Lung Cancers

**DOI:** 10.3389/fcell.2021.677860

**Published:** 2021-07-21

**Authors:** Ke Li, Xinling Zhu, Conghu Yuan

**Affiliations:** ^1^Department of Oncology, Jiangsu Cancer Hospital, Jiangsu Institute of Cancer Research, The Affiliated Cancer Hospital of Nanjing Medical University, Nanjing, China; ^2^Department of Operating Room, Jiangsu Cancer Hospital, Jiangsu Institute of Cancer Research, The Affiliated Cancer Hospital of Nanjing Medical University, Nanjing, China; ^3^Department of Anesthesiology, Yancheng Third People’s Hospital, The Yancheng School of Clinical Medicine of Nanjing Medical University, Yancheng, China

**Keywords:** lung cancer, miR-185-3p, PFKL, ER-resistance, tyrosine kinase inhibitor

## Abstract

Erlotinib (ER), as an epidermal growth factor receptor (EGFR) tyrosine kinase inhibitor (TKI), has a significant therapeutic effect in lung cancers. However, EGFR TKI resistance inevitably occurs after treatment for approximately 12 months, which weakens its antitumor effect. Here, we identified miR-185-3p as a significantly downregulated microRNA responsible for acquired EGFR TKI resistance in cells and patients with lung cancer. qRT-PCR and Western Blot were performed to determine the relative expression of miR-185-3p in ER-resistant tumor tissues and cells. The viability and apoptosis of lung cancer cells were evaluated by Cell Counting Kit-8 (CCK8) assay and flow cytometry, respectively. The binding between miR-185-3p and liver-type phosphofructokinase (PFKL) was verified by dual luciferase assay. It was found that overexpression of miR-185-3p conferred ER sensitivity in lung cancer cell lines. MiR-185-3p was downregulated in ER-resistant lung cancer cells (H1299/ER and A549/ER). MiR-185-3p inhibited proliferation and induced cell apoptosis in ER-resistant cells. Mechanistically, miR-185-3p downregulation contributed to ER resistance through upregulating the PFKL. Moreover, Mesenchymal to epithelial transition (MET) oncoprotein promoted EGFR-TKI resistance by regulating miR-185-3p and PFKL. These findings revealed a novel mechanism in which downregulation of miR-185-3p may induce overexpression of PFKL and MET and confer ER resistance in lung cells. Combination of PFKL/MET inhibitors and EGFR TKIs could be a rational therapeutic approach for lung cancer patients with EGFR mutation.

## Introduction

Lung cancer is a main cause of death worldwide. There are about 2 million newly diagnosed cases of lung cancer and 1.8 million deaths each year (Chen Y. et al., 2020). The 5-years survival rate for patients with non-small-cell lung cancer is no more than 16% (Fu et al., 2020). For lung adenocarcinoma, the median survival period is 4–5 months and the 1-year survival rate is less than 10% (Luo et al., 2019). Clinically, surgery, radiotherapy, and chemotherapy are the main treatments for lung cancers (Pan et al., 2019). Targeted drugs are used in most lung cancer patients who are diagnosed at advanced stages and infeasible for surgery (Pdq Cancer Information Summaries, 2002; Xia et al., 2018). Erlotinib (ER), an epidermal growth factor receptor (EGFR) tyrosine kinase inhibitor (TKI), is a targeted drug for lung cancer treatment. EGFR TKI has been successfully employed in the clinic, especially in lung cancer patients who have EGFR mutations (Capelletto and Novello, 2012; Suda et al., 2012). However, it is inevitable that the vast majority of patients with ER treatment become resistant within 9–13 months, which weakens its antitumor effect (Camidge et al., 2014; van der Wekken et al., 2016). Therefore, it is necessary to investigate the mechanism of ER resistance in order to elevate the treatment effect of lung cancer patients.

MicroRNAs (miRNAs) play vital roles in lung cancer progression (Hu et al., 2018; Wang et al., 2018). It is well known that miRNAs can affect mRNA level to promote or inhibit tumor progression, such as cell proliferation, apoptosis, epithelial–mesenchymal transition (EMT), and metastasis (Li S. et al., 2018; Lu C. et al., 2018). Abnormal expression of miR-185-3p is found in various tumors. For example, miR-185-3p was suppressed in both colorectal cancer (Zhou C. et al., 2020) and breast cancer (Lu G. et al., 2018). MiR-185-3p inhibited the invasion and metastasis of nasopharyngeal carcinoma *via* WNT2B (Liu et al., 2017). A growing body of evidence showed that miRNAs were associated with ER resistance (Zheng et al., 2020). For example, MiR-124 affects ER resistance in pancreatic cancer by targeting EphA2 (Du et al., 2019). By repressing the level of neurofibromatosis 1, the upregulated expression of miR-641 promotes resistance to ER in non-small-cell lung cancer cells (Chen et al., 2018). Previous studies indicated that miR-185-3p can promote the antitumor effect of other antitumor drugs (Uhr et al., 2019; Zhou C. et al., 2020). However, it is not clear whether miR-185-3p affects the ER resistance in lung cancer. Phosphofructokinase (PFK) is a critical rate-limiting enzyme that promotes the phosphorylation of fructose-6-phosphate to fructose-1,6-bisphosphate, a core step in the pathway of glycolysis (Wegener and Krause, 2002). A recent study found that repression of PFK suppressed cell proliferation and oncogenicity (Yi et al., 2012). There are three subtypes of PFK in human: PFKL (liver), PFKM (muscle), and PFKP (platelet) (Yalcin et al., 2009; Lee et al., 2017). However, the role of PFKL in lung cancer cells is unknown.

Activation of alternative or bypass pathway often causes primary drug resistance. Through activation of bypass pathway, cancer cells can survive and proliferate, even when they are inhibited by the initial driver pathway. The most common bypass pathway is MET amplification, which accounts for 5–10% of cases with acquired resistance to EGFR TKIs (Wu and Shih, 2018). MET gene amplification could activate phosphoinositide 3-kinase (PI3K)–AKT signaling pathway, which is independent of EGFR, through driving Erb-B2 receptor tyrosine kinase 3 (ERBB3) dimerization and signaling. However, the threshold of MET amplification that would induce TKI resistance has not been clarified. Overexpression of hepatocyte growth factor, the ligand of MET oncoprotein, also promotes EGFR TKI resistance. Activation of other alternative pathways, including human epidermal growth factor receptor 2 (HER2) amplification, phosphatidylinositol-4,5-bisphosphate 3-kinase catalytic subunit alpha (PIK3CA) mutation, v-raf murine sarcoma viral oncogene homolog B1 (BRAF) mutation, and increased expression of the receptor tyrosine kinase AXL, has been reported to promote acquired resistance to EGFR TKIs (Wu and Shih, 2018).

In this study, we aimed to investigate the role of miR-185-3p in lung cancer progression and ER resistance. Lung cancer cell lines were used to evaluate the function of miR-185-3p on lung cancer and the association among miR-185-3p, PFKL, and MET. Our results indicated that PFKL/MET inhibitors in combination with EGFR TKIs could be synergistic in the clinical management of lung cancer.

## Materials and Methods

### Patients

Tumor tissues of ER-resistant (*n* = 30) and ER-sensitive (*n* = 30) lung cancer were obtained from patients who underwent surgery at the Nanjing Medical Hospital. Normal tissues adjacent to cancers were obtained at the same time. All tissues were kept frozen until used. The 5-years overall survival was assessed with ER treatment until death. The collection of clinical samples was approved by the Ethics Committee of Nanjing Medical University. All patients have signed their informed consents.

### Cell Culture and Erlotinib Treatment

Human lung cancer cell lines, H1299 and A549, were obtained from Procell Life Science and Technology (Wuhan, China). The cell lines were maintained in RPMI-1640 (Procell Life Science and Technology, Wuhan, China) containing 10% fetal bovine serum (FBS; Procell Life Science and Technology, Wuhan, China), penicillin (100 U/ml), and streptomycin (50 g/ml) in a humidified CO_2_ incubator at 37°C. ER-resistant cell lines (H1299/ER, A549/ER) were induced by treatment with ER (HY-50896, MedChemExpress, Shanghai, China) (Wu et al., 2020). Briefly, the ER-resistant cell lines were developed from H1299 and A549 cells by stepwise exposure to increasing concentrations of ER from 2 nM to 10 μM, and the drug-resistant cell lines H1299/ER and A549/ER were established after 3 months.

### Cell Transfection

Si-MET, si-NC, NC mimic, miR-185-3p mimic, Vector, and PFKL plasmid were obtained from RIBOBIO (Guangzhou, China). A549/ER and H1299/ER cells were seeded in six-well plates at a density of 6 × 10^4^ and 3.5 × 10^5^ cells/well, respectively. Transfections were performed using Lipofectamine 2000 (Invitrogen, CA, United States) following the manufacturer’s instructions.

### RNA Extraction and qRT-PCR Assay

Total RNA was extracted from tissues and cell lines using TRIzol reagents (R1030, Applygen, Beijing, China). Reverse transcription was performed by the Applied Biosystems High-Capacity cDNA Reverse Transcription Kit (D1802, Haigene, Harbin, China) for miR-185-3p and Kit (D0401, Haigene, Harbin, China) for PFKL. Quantitative RT-PCR was performed with the ABI PRISM 7500 using TaqMan primers, probes for PFKL, and control gene β-actin (Thermo Fisher) (Zhao et al., 2017). The primers are listed in Table 1. U6 (Zhou C. et al., 2020) and β-actin (Li L. et al., 2018) act as endogenous controls. Samples were analyzed with the comparative CT method, where fold change was calculated from the ΔΔCT value with the formula 2^–ΔΔ*Ct*^.

**TABLE 1:** Primer sequences for qRT-PCR.

Primer name	(5′–3′) Primer sequences
miR-185-3p-Forward	5′-GGGGCTGGCTTTCCTCTG-3′
miR-185-3p-Reverse	5′-GTGGAGTCGGCAATTGCAC−3′
U6-Forward	5′-CTCGCTTCGGCAGCACA−3′
U6-Reverse	5′-AACGCTTCACGAATTTGCGT−3′
PFKL-Forward	5′-GTGGTTGTCGGAGAAGCTGCGC−3′
PFKL-Reverse	5′-CGGTGCTCGAAATCAGTGTCT−3′
β-actin-Forward	5′-GACCTGACTGACTACCTCATGAAGAT−3′
β-actin-Reverse	5′-GTCACACTTCATGATGGAGTTGAAGG−3′

### Cell Viability

Cell viability was assessed with CCK8 kit (HY-K0301, MedChemExpress, Shanghai, China). A549 and H1299 cells were cultured at a density of 10^4^/well. Followed by incubating with 10 μl CCK8 for 1–4 h. The light absorbance was measured by GloMax System (Promega, WI, United States) at 450 nm wavelength (Chen C. et al., 2020). The percentage of growth was determined and compared to that of the untreated controls.

### Apoptosis Detection

The apoptotic rate of lung cancer cells was evaluated by Annexin V-fluorescein isothiocyanate (FITC)/propidium iodide (PI) Apoptosis Detection Kit (40302ES20, Yeasen, Shanghai, China) *via* flow cytometry. A549/ER and H1299/ER cells were seeded in six-well plates at a density of 6 × 10^4^ and 3.5 × 10^5^ cells/well, respectively. When cell confluence reached 80%, cells were harvested, followed by staining with 10 μl of Annexin V-FITC and PI. Then, cells were analyzed by a flow cytometer (BD, New Jersey, United States) (Wang et al., 2020).

### Western Blot

Tissues or cells were washed with phosphate buffered saline (PBS) three times and lysed in radioimmunoprecipitation assay (RIPA) lysis buffer (Cell Signaling Technology, Shanghai, China), containing both protease and phosphatase inhibitors (APExBIO, Houston, United States). Pierce bicinchoninic acid (BCA) protein assays (Thermo Scientific, United States) were used to determine protein concentrations. Protein was resolved on 10% sodium dodecyl sulfate polyacrylamide gel electrophoresis (SDS-PAGE) gels, transferred onto polyvinylidene fluoride (PVDF) membranes, and blocked at 37°C for 1 h, followed by incubation with primary antibodies: rabbit anti-BAX (1:5,000, ab32503), anti-Bcl-2 (1:1,000, ab59348), anti-Cleaved-caspase3 (1:1,000, ab49822), anti-Cleaved-caspase9 (1:1,000, ab2324), anti-PFKL (1:5,000, ab181064), anti-MET, anti-HER2, anti-AXL, anti-ERBB3, anti-PI3K, and rabbit anti-β-actin (1:2,000, ab8227) at 4°C overnight. The membranes were then re-probed with immunoglobulin G (IgG) (1:2,000, ab6721) antibody. The immune reactive bands were visualized by ECL Prime Western Blotting Detecting Reagent and exposed using a Chemiluminescence Western Blot Scanner (CDIGIT). The integrated density of each band was quantified by ImageJ software (ImageJ Software Inc., United States). Antibodies mentioned above were supplied by Abcam (Cambridge, United Kingdom).

### Dual Luciferase Reporter Assay

PFKL-WT (containing the binding sites of miR-185-3p at PFKL 3′-UTR) and PFKL-MUT (mutation of binding sites) were cloned into Luciferase Reporter Vector (AM5795, Invitrogen, CA, United States). PFKL-WT or PFKL-MUT was co-transfected with miR-185-3p mimic into H1299/ER or A549/ER cells. After transfection for 48 h, H1299/ER and A549/ER cells were evaluated using the Reporter Vector System (AM5795, Invitrogen, CA, United States) using a GloMax 20/20 Luminometer (Promega, WI, United States) (Zhang et al., 2020).

### RNA Pull-Down Assay

The miR-185-3p mimic, miR-185-3p mock, and their control were labeled with different biotins, Bio-miR-185-3p WT, Bio-miR-185-3p MUT, or Bio-NC. A PureBinding^TM^ RNA–Protein Pull down Kit (P0201, Geneseed, Guangzhou, China) was employed to carry out the RNA pull-down assay. Briefly, biotin-labeled miR-185-3p was incubated with streptavidin agarose beads (Invitrogen, CA, United States) for 1 h. Finally, the pulled-down PFKL was detected by qRT-PCR (Wang and Chang, 2020).

### Statistical Analysis

The mean ± SD represents data from three replicates. SPSS version 22.0 software (IBM Corp., NY, United States) was applied for statistical analysis of all data, and Prism version 7.0 (GraphPad) was used to generate graphics. Student’s *t*-test was performed for comparison between two groups. One-way ANOVA and Tukey’s posttests were used for multiple groups. The level of significance was *p* < 0.05.

## Results

### The Correlation of miR-185-3p With Erlotinib Resistance in Erlotinib-Resistant Cells and Tissues

**FIGURE 1:**
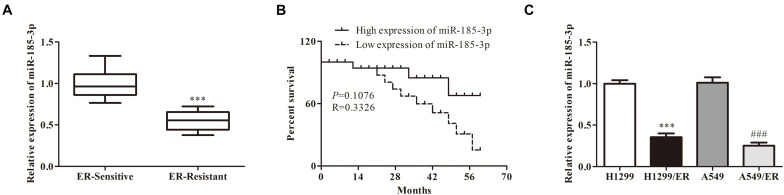
The correlation of miR-185-3p with erlotinib (ER) resistance in ER-resistant cells and tissues. **(A)** The relative expression of miR-185-3p was determined by qRT-PCR in ER-sensitive and ER-resistant tissues. **(B)** The survival rate of patients with different levels of miR-185-3p. **(C)** The relative expression of miR-185-3p was determined by qRT-PCR in various lung cancer cell lines. Data were shown as mean ± SD. ^∗∗∗^*p* < 0.001; ###*p* < 0.001.

To evaluate the role of miR-185-3p in ER-resistant lung cancer patients, the level of miR-185-3p was evaluated by qRT-PCR in ER-resistant and ER-sensitive tissues. The data revealed that miR-185-3p was suppressed in ER-resistant tissues compared with ER-sensitive tissues (Figure 1A). Then, we assessed the relationship between miR-185-3p level in tumor tissues and the survival data. The result demonstrated that the high level of miR-185-3p was associated with a high survival rate (Figure 1B). In the *in vitro* cell models, the level of miR-185-3p in H1299/ER and A549/ER cells was significantly lower than those in H1299 and A549 cells, respectively (Figure 1C). These results suggested that miR-185-3p is downregulated in ER-resistant lung cancer cells and tissues. The level of miR-185-3p is correlated with patients’ survival.

### MiR-185-3p Suppressed Proliferation and Induced Apoptosis of Lung Cancer Cells

**FIGURE 2:**
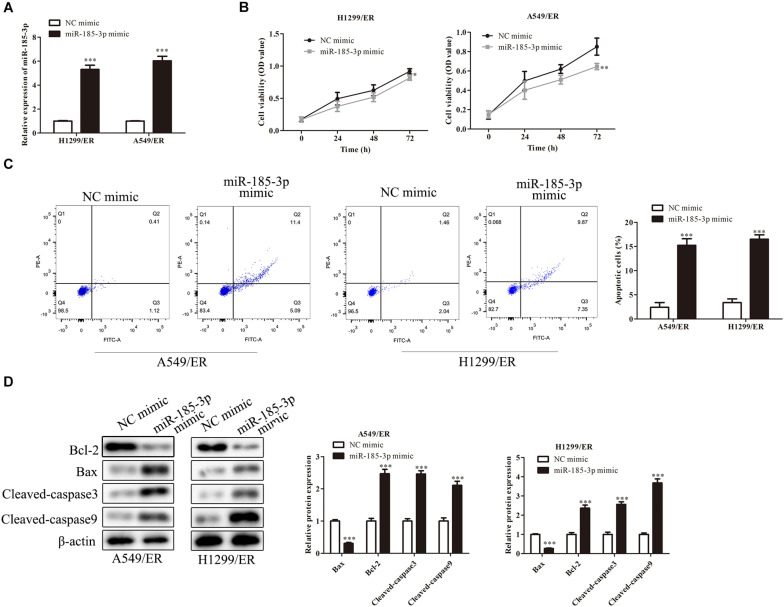
MiR-185-3p suppresses cell proliferation and induces apoptosis. **(A)** The level of miR-185-3p was determined by qRT-PCR in H1299/ER and A549/ER cells. **(B)** Cell viability was determined by Cell Counting Kit-8 (CCK8) assay in H1299/ER and A549/ER cells at 24, 48, and 72 h. **(C)** Cell apoptosis was determined by flow cytometry assay in H1299/ER and A549/ER cells. **(D)** The levels of apoptosis-related proteins Bcl-2, Bax, Cleaved-caspase3, and Cleaved-caspase9 were investigated by Western blot in H1299/ER and A549/ER cells. Data were shown as mean ± SD. *p < 0.05; ^∗∗^*p* < 0.01; ^∗∗∗^*p* < 0.001.

To further verify the role of miR-185-3p on lung cancer cells, we overexpressed miR-185-3p with miR-185-3p mimic in both H1299/ER and A549/ER cells. The level of miR-185-3p increased in both H1299/ER and A549/ER cells treated with miR-185-3p mimic (Figure 2A). Moreover, the cell viability was significantly suppressed in both H1299/ER and A549/ER cells treated with miR-185-3p mimic for 72 h (Figure 2B). Besides, cell apoptosis was markedly induced by miR-185-3p mimic in H1299/ER and A549/ER cells (Figure 2C). To further verify the level of apoptosis, the level of apoptosis-related proteins was investigated by Western blot. The data demonstrated that Bax was suppressed, whereas Bcl-2, Cleaved-caspase3, and Cleaved-caspase9 were markedly promoted by miR-185-3p mimic (Figure 2D). These results demonstrated that miR-185-3p could suppress cell proliferation and induce cell apoptosis.

### MiR-185-3p Targeted Liver-Type Phosphofructokinase and Downregulated Its Expression

**FIGURE 3:**
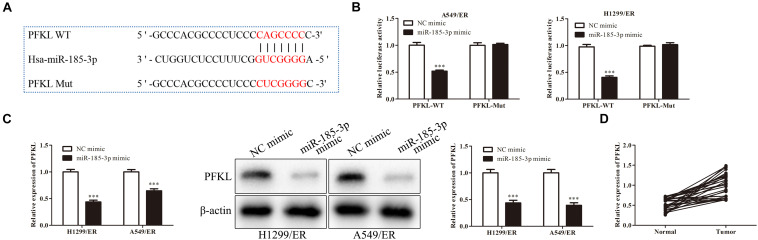
MiR-185-3p targets liver-type phosphofructokinase (PFKL) and downregulates its expression. **(A)** The bioinformatics analysis of miR-185-3p and PFKL. **(B)** MiR-185-3p mimic targeting PFKL was determined by dual luciferase reporter assay. **(C)** The level of PFKL in H1299/ER and A549/ER cells was detected by Western blot. **(D)** Spearman’s correlation analysis assessed the PFKL expression in normal and tumor tissues. Data were shown as mean ± SD. ^∗∗∗^*p* < 0.001.

Liver-type phosphofructokinase (PFKL) was predicted to be a target of miR-185-3p *via* bioinformatics analysis (Figure 3A) and confirmed by dual luciferase reporter assay. MiR-185-3p mimic and PFKL-WT or PFKL-Mut were co-transfected into H1299 cells. Dual luciferase reporter assay showed that miR-185-3p mimic inhibited the luciferase activity of PFKL-WT in A549 cells (Figure 3B). To further demonstrate the interaction between miR-185-3p and PFKL, the expression of PFKL was examined by qRT-PCR and Western blot. The data revealed that PFKL was markedly inhibited by miR-185-3p mimic in both H1299/ER and A549/ER cells (Figure 3C), demonstrating that miR-185-3p targeted PFKL and downregulated its expression. Next, qRT-PCR was carried out to assess PFKL expression in normal and tumor tissues. An inverse correlation was observed *via* Spearman’s correlation analysis, indicating that PFKL level was higher in normal tissues than that in tumor tissues (Figure 3D).

### Liver-Type Phosphofructokinase Was Upregulated in Erlotinib-Resistant Cells and Tissues

**FIGURE 4:**
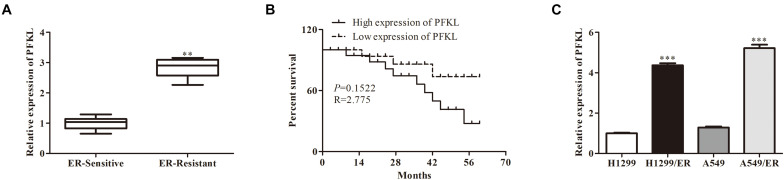
Liver-type phosphofructokinase was upregulated in ER-resistant cells and tissues. **(A)** The relative expression of PFKL was determined by Western blot in ER-sensitive and ER-resistant tissues. **(B)** The survival rate of patients with different levels of PFKL. **(C)** The relative expression of PFKL was determined by Western blot in various lung cancer cell lines. Data were shown as mean ± SD. ^∗∗^*p* < 0.01; ^∗∗∗^*p* < 0.001.

Analysis with qRT-PCR revealed that PFKL level was higher in ER-resistant tissues than in ER-sensitive tissues (Figure 4A). The low expression of PFKL was associated with high survival rate of patients (Figure 4B). Besides, the levels of PFKL in H1299/ER and A549/ER cells were significantly higher than those in H1299 and A549 cells. The above results demonstrated that PFKL was overexpressed in ER-resistant cells (Figure 4C).

### MiR-185-3p Downregulated Liver-Type Phosphofructokinase to Inhibit Tumor Cell Proliferation and Erlotinib Resistance

**FIGURE 5:**
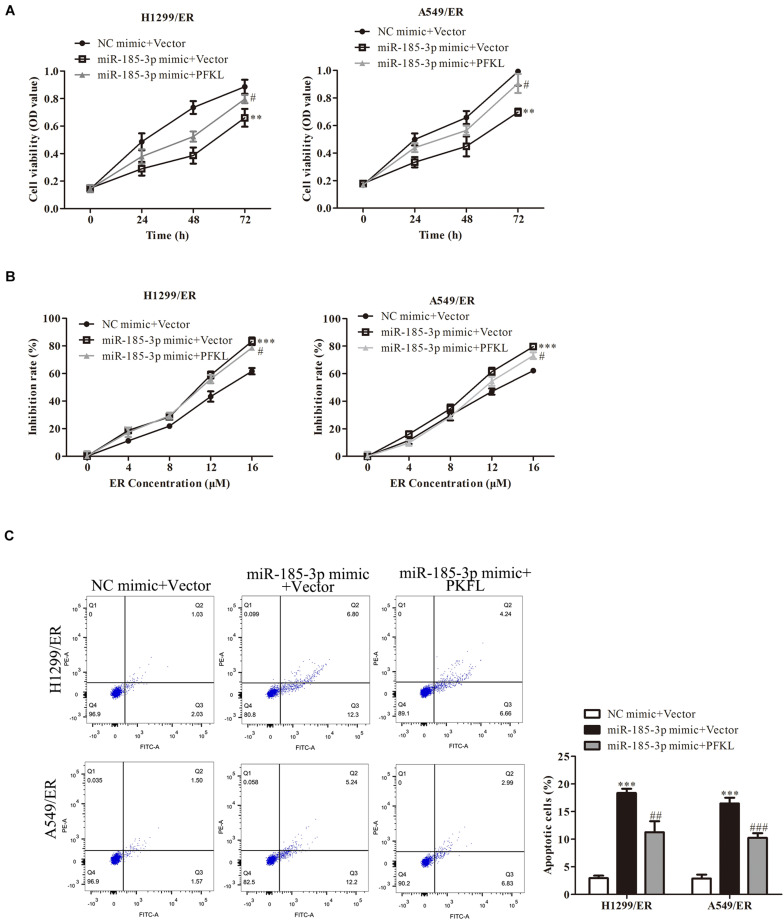
Liver-type phosphofructokinase inhibits tumor cell proliferation and ER resistance. **(A)** Cell viability was determined by CCK8 assay in H1299/ER and A549/ER cells at 24, 48, and 72 h. **(B)** The inhibition rate was determined with various ER concentrations from 0 to 16 μM in H1299/ER and A549/ER cells. **(C)** Cell apoptosis was determined using flow cytometry assay in H1299/ER and A549/ER cells. Data were shown as mean ± SD. ^∗∗^*p* < 0.01; ^∗∗∗^*p* < 0.001 vs. NC mimic + Vector, #*p* < 0.05, ###*p* < 0.001 vs. miR-185-3p mimic + Vector.

The role of PFKL under miR-185-3p regulation in lung cancer cells was assessed. We co-transfected miR-185-3p mimic and PFKL plasmids into both H1299/ER and A549/ER cells. The data depicted that cell viability was strikingly suppressed by miR-185-3p mimic, whereas that was reversed by PFKL overexpression (Figure 5A). The cell growth inhibitory rate in both H1299/ER and A549/ER cells increased with increasing ER concentration from 0 to 16 μM. Moreover, the inhibitory rate of miR-185-3p mimic in both H1299/ER and A549/ER cells was higher than that in cells transfected with NC mimic, whereas that was reversed by PFKL overexpression (Figure 5B). Besides, cell apoptosis was increased by miR-185-3p mimic, whereas it was alleviated by PFKL overexpression (Figure 5C). These results indicated that miR-185-3p suppressed cell proliferation and induced cell apoptosis by downregulating PFKL.

### MiR-185-3p/Liver-Type Phosphofructokinase Alleviates Erlotinib Resistance of Lung Cancer Cells by Inhibiting Epidermal Growth Factor Receptor Alternative Pathway

**FIGURE 6:**
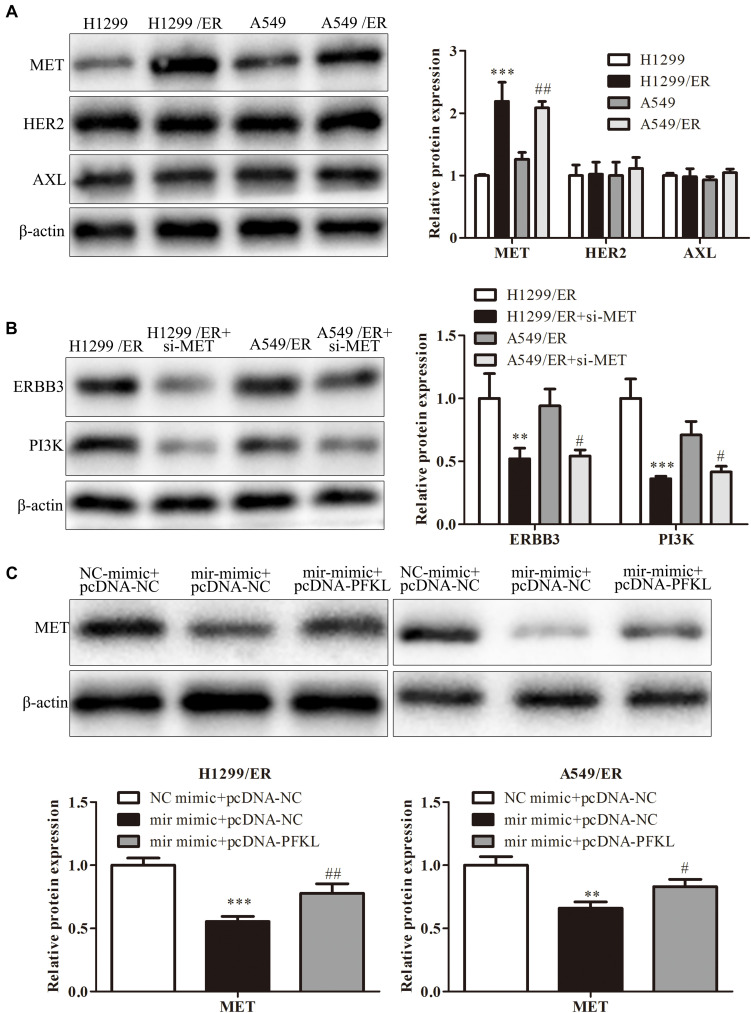
MiR-185-3p/PFKL alleviates ER resistance of lung cancer by inhibiting the epidermal growth factor receptor (EGFR) alternative pathways. **(A)** The levels of MET, human epidermal growth factor receptor 2 (HER2), and AXL were determined by Western blot in H1299, A549, H1299/ER, and A549/ER cells. **(B)** The levels of Erb-B2 receptor tyrosine kinase 3 (ERBB3) and phosphoinositide 3-kinase (PI3K) were determined by Western blot in H1299, A549, H1299/ER, and A549/ER cells with or without si-MET. **(C)** The level of MET was determined by Western blot in H1299/ER and A549/ER cells. Data were shown as mean ± SD. ^∗∗^*p* < 0.01; ^∗∗∗^*p* < 0.001; #*p* < 0.05; ##*p* < 0.01.

Erlotinib could stimulate tumor cell to produce resistance by alternative pathways such as MET, HER2, or AXL pathway. Western blot was used to examine the expression of molecules in these pathways. The findings revealed that the level of MET in H1299/ER and A549/ER cells was strikingly higher than that in H1299 and A549 cells, while the level of HER2 and AXL remained unchanged (Figure 6A). These results illuminated that ER resistance might be caused by activation of the MET signaling pathway. Furthermore, the levels of ERBB3 and PI3K were markedly decreased in both H1299/ER and A549/ER cells transfected with si-MET compared with cells transfected with si-NC (Figure 6B), indicating that MET could activate EGFR downstream signaling molecules, such as ERBB3 and PI3K. The MET level of treatment with miR-185-3p mimic in both H1299/ER and A549 cell lines was lower than that of NC mimic, whereas that was reversed by PFKL overexpression. These results demonstrated that miR-185-3p/PFKL could regulate the activation of the MET alternative pathway in ER-resistant lung cancer cells (Figure 6C).

## Discussion

The development of lung cancer is a multistep process involved in the accumulation of genetic and epigenetic changes that lead to DNA damage and eventually the conversion of epithelial cells into cancer cells (Asghariazar et al., 2019). Lung cancer has become one of the most common cancers because of lifestyle changes and increased risk factors (Hosseini et al., 2009).

MicroRNAs are short non-coding RNAs that affect the expression of target genes by inhibiting their translation or degrading their messenger RNAs (Yang et al., 2020). There is growing body of evidence indicating that miRNAs play different roles in the progression of lung cancers. MiR-222-3p stimulates the proliferation and represses apoptosis of non-small-cell lung cancer cells *via* repressing p53 upregulated modulator of apoptosis (PUMA) (Chen and Li, 2020). A study conducted by Li and Yuan (2020) revealed that miR-103 was elevated in A549 and H1299 cells and promoted cell growth and EMT and reduced cell apoptosis. On the contrary, miR-330 represses the viability, proliferation, and migration of lung cancer cells (Mohammadi et al., 2020). Another study conducted by Wei et al. (2020) revealed that miR-16 represses the growth and metastasis of lung cancer cells by suppressing Yes-associated protein 1 (YAP1) level. In this study, our data demonstrated that miR-185-3p was downregulated in lung cancer tissues, repressed cell proliferation, and stimulated cell apoptosis.

Although their definite role in targeted treatment is not yet clear, miRNAs can alter tumor sensitivity to antitumor drugs (Liu et al., 2020). A number of studies have shown that some antitumor drugs exert their antitumor effect *via* affecting miRNAs and their targeted genes. Chikuda et al. (2020) revealed that miR-629-3p is associated with conferring anticancer drug resistance in head and neck cancer. Kaempferol was demonstrated to play an anticancer role, which attenuated oxygen glucose deprivation (OGD)-induced cell damage *via* suppressing miR-15b level stimulated by the PI3K/AKT and Wnt3a/β-catenin signaling pathways (Li et al., 2020). ER, as an EGFR TKI, was demonstrated to play the anticancer role in cancer treatment. MiR-23a suppression elevated the ER sensitivity of lung cancer stem cells *via* PTEN/PI3K/Akt signaling pathway (Han et al., 2017). miR-223 level was markedly enhanced in HCC827/ER cells, and suppressing miR-223 led to alleviated resistance in HCC827/ER cells (Zhang et al., 2017). On the contrary, our data demonstrated that miR-185-3p was downregulated in ER-resistant lung cancer tissues and cells, and miR-185-3p reduced ER resistance in lung cancer cells.

To investigate the mechanism of miR-185-3p in ER resistance, we identified that PFKL is a putative target of miR-185-3p by bioinformatics analysis and verified by dual luciferase reporter assay. PFKL is one of the subtypes of PFK in human (Lee et al., 2017). In hepatocellular carcinoma (HCC), PFKL was degraded by interaction with A20 and suppressed the progression of HCC (Feng et al., 2020). Iodine-125 irradiation played an anticancer role in HCC by stimulating miR-338/PFKL axis (Zheng et al., 2019). PFKL could be upregulated by TAp73 and then enhance cell proliferation and tumor growth (Li L. et al., 2018). Yang et al. (2016) revealed that PFKL is suppressed by miR-128 and stimulates lung cancer cell growth *in vitro*. Similarly, our data revealed that miR-185-3p targeted PFKL to repress cell proliferation and ER resistance in lung cancer.

In summary, our findings elucidated that miR-185-3p was suppressed and PFKL was elevated in ER-resistant lung cancer tissues and cells. MiR-185-3p repressed the level of PFKL/MET to repress cell proliferation and ER resistance in lung cancer. Therefore, targeting the miR-185-3p/PFKL/MET axis may serve as a potential treatment for ER-resistant lung cancer.

## Data Availability Statement

The original contributions presented in the study are included in the article/supplementary material, further inquiries can be directed to the corresponding author.

## Ethics Statement

The studies involving human participants were reviewed and approved by the Ethics Committee of Nanjing Medical University. The patients/participants provided their written informed consent to participate in this study.

## Author Contributions

CY conceived the study and obtained the funding. KL and XZ performed the experiments and analyzed the data. All authors participated in writing and revising the article and approved the final manuscript.

## Conflict of Interest

The authors declare that the research was conducted in the absence of any commercial or financial relationships that could be construed as a potential conflict of interest.
